# Comparison of Stimulant-Related Presentations to Victorian Emergency Departments Pre-pandemic and During the COVID-19 Pandemic

**DOI:** 10.7759/cureus.28813

**Published:** 2022-09-05

**Authors:** Peter T Redona, Cindy Woods, Debra Jackson, Jane Hayman, Kim Usher

**Affiliations:** 1 Faculty of Health and Medicine, University of New England, Armidale, AUS; 2 Substance Use and Addiction, Health Research Institute, University of Canberra, Canberra, AUS; 3 Faculty of Health and Medicine, University of Sydney, Sydney, AUS; 4 Epidemiology and Public Health, Accident Research Centre, Monash University, Clayton, AUS

**Keywords:** pandemic, methamphetamine, emergency departments, cocaine, amphetamine

## Abstract

Introduction

Victoria, Australia, holds the unenviable record for the longest number of lockdown days in the world (262 days) and some of the most rigid restrictions. The purpose of this study was to determine whether changes in harmful drug use occurred during the pandemic by comparing stimulant-related presentations to Victorian emergency departments before and during the COVID-19 pandemic.

Methods

A retrospective analysis of data from the Victorian Injury Surveillance Unit was undertaken for two time periods, March 2019 to September 2019 and March 2020 to September 2020.

Results

The proportion of people presenting to an ED who used methylamphetamine/methamphetamine/amphetamine significantly increased from 2019 to 2020. Conversely, there was a significant reduction in ED presentations among people who used 3,4-methylenedioxy​methamphetamine (MDMA) and ecstasy during the study period.

Conclusions

COVID-19-related restrictions can affect mental health due to depression, or anxiety, particularly if people also experience loss of employment and income. In addition, mental health issues may affect substance use, including increased frequency of use and dose. This has implications for policy and planning during a pandemic and may be overlooked as the focus is on planning and resources for patients with COVID-19.

## Introduction

The COVID-19 pandemic affected the way Victorian people access, supply, and market illicit drugs due to the imposed restriction of movement, social distancing, isolation measures, and restrictions on mass gatherings [1]. In addition, historical events demonstrate changes in drug use and harm brought about by rapid disruption in the market and drug supply [1]. An example is the heroin shortage in 2001, characterized by a decline in the availability and purity of heroin, causing a significant change in the use of heroin in Australia [2].

Price O et al. (2021) analyzed the Ecstasy and Related Drugs Reporting System (EDRS) in Australia [1]. They found that the COVID-19 pandemic had little impact on access to stimulants, e.g., ecstasy, 3,4-methylenedioxy​methamphetamine (MDMA), and related stimulants. Participants in the study reported a decrease in the frequency of use of stimulants and related drugs, namely cocaine and ketamine, relative to pre-COVID-19 with its associated restrictions. However, several participants noted an increase in the frequency of their substance use, which was most likely brought about by social reasons, e.g., boredom and isolation [2]. The report noted that the median price of MDMA capsules decreased while the price of other drugs, e.g., cannabis, remained stable [2]. The report claims that the perceived purity of ecstasy and MDMA remained stable throughout the pandemic [2].

Various international studies reported changes in drug usage and related harms brought about by the pandemic. According to the European Monitoring Centre for Drugs and Drug Addiction (EMCDDA), disruptions in supply chains across Europe contracted the drug market but only to a small-to-moderate extent, as evidenced by the reduced number and volume of seizures at the country's borders [3]. The EMCDDA also claims a reduction of drug-related offenses as ensuring public safety and order became the priority of the police systems [3]. In America, mortality rates related to drug overdose were higher after the onset of COVID-19 compared to the months before the pandemic in 2019 [4]. The purpose of this study is to determine whether changes in harmful drug use occurred during the pandemic by comparing stimulant-related presentations to Victorian emergency departments before and during the COVID-19 pandemic.

## Materials and methods

Study design and procedure

A population-based retrospective analysis of data from the Victorian Injury Surveillance Unit (VISU) was undertaken describing stimulant-related ED presentations before and during the COVID-19 pandemic from March 2019 to September 2019 and March 2020 to September 2020.

Setting

Victoria is Australia's second most populated state, second only to New South Wales, with an estimated population of 6.64 million people by June 2021 [5]. Victoria was hit with two waves of COVID-19 and experienced the longest and most stringent lockdowns in the world as strict social distancing measures, and curfew policies were applied by the Victorian Government [6]. Also, according to the Australian Bureau of Statistics, in June 2021, at least 27% of people living in Victoria experienced high or very high levels of psychological distress. This is higher when compared with the rest of Australia (18%) [7].

Victorian Injury Surveillance Unit (VISU)

The VISU analyzes, interprets, and disseminates data on injury deaths, hospital admissions, and ED presentations in the state of Victoria [8]. ED presentation data are supplied by the Victorian Department of Health and collated in the Victorian Emergency Minimum Dataset (VEMD). Currently, 39 Victorian hospitals contribute to the VEMD [9].

Data analysis

Presentations in the VEMD have a free-text narrative section that provides further detail about the injury event. Stimulant drug types were selected using a combination of the International Statistical Classification of Diseases and Related Health Problems, Tenth Revision, Australian Modification (ICD10-AM) diagnosis codes, and a text search of the narrative 'Description of Event' field. The ICD10-AM diagnosis codes and keywords used to select cases are shown in Table 1 for all drug types. All cases were manually checked for relevance.

**Table 1:** Search method for stimulant-related presentations. *Cases related to speed were not reported due to small cell counts in both years. ^Note some cases for ecstasy/MDMA were found to have the diagnosis code 'T43.69'. #All cases with 'coke' in the text field related to the drink coca cola.

Drug name	ICD10-AM Diagnosis code	And/Or Key terms
Speed*	N/A	‘speed’ + cause was poisoning
Ice/Crystal meth	T43.69' Other psychostimulants with potential for use disorder	‘ice’, ‘crystal meth’, ‘crystalmeth’ ‘chrystal meth’, ‘chrystalmeth’
Methamphetamine/ Metamphetamine/ Methylamphetamine	T43.69' Other psychostimulants with potential for use disorder	‘methylamphetamine’, ‘metamphetamine’, ‘methamphetamine', 'metamphetamine’, ‘amphetamine’
Ecstasy/MDMA	‘T4362’ poisoning by Methylenedioxy-methamphetamine/ecstasy/MDMA ‘T43.69’ ‘Other psychostimulants with potential for use disorder’^	‘mdma’, ‘ecstacy’, ‘xtc’, ‘exstacy’, ‘exstasy’, ‘extasy’, ‘extacy’, ‘ectasy’
Cocaine	‘T40.5’ poisoning by narcotics and psychodysleptics [hallucinogens] - cocaine	‘cocaine’, ‘coke’#

Data were descriptively analyzed using z-tests for two population proportions to examine changes between March to September 2019 and 2020 [10].

## Results

**Figure 1:**
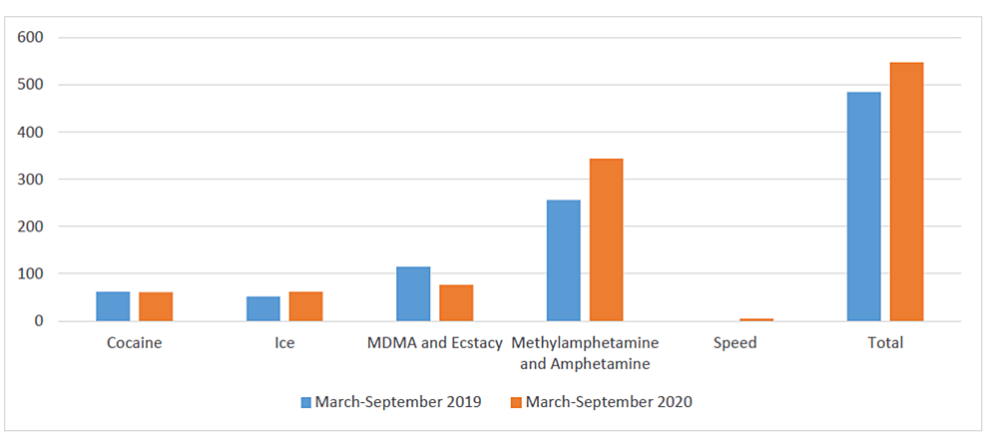
ED presentations of people who use stimulants to Victorian hospitals.

Table 2 summarizes stimulant-related ED presentations to Victorian hospitals from March 2019 to September 2019 and March 2020 to September 2020. The most significant increase (34%) can be noted in the ED presentations of people who used methylamphetamine/methamphetamine/amphetamine. Conversely, there was a marked reduction (34%) in ED presentations of people who used MDMA and ecstasy. However, the COVID-19 pandemic and associated restrictions did not cause any significant difference in the ED presentations of people who used cocaine or ice. Numbers for speed were too low to report for reasons of confidentiality. There was a total of 485 stimulant-related ED presentations in 2019 versus 543 in 2020 (Table 2 and Figure 1).

**Table 2:** Z-test analysis of ED presentation of people who used stimulant pre-COVID-19 and during COVID-19 lockdown.

Stimulant type	March-September 2019	March-September 2020	Z-Score	P-value
Cocaine	62	61	0.764	0.447
Ice	52	62	-.335	0.726
MDMA and Ecstasy	115	76	3.998	< 0.001
Methylamphetamine/Methamphetamine/Amphetamine	256	344	-3.431	< 0.001
Total	485	543		

## Discussion

This is one of the few Australian studies to provide state-wide data on stimulant-related ED presentations across Victoria. Overall there were no significant changes in the harmful use of cocaine or ice pre and during the pandemic. However, ED presentations for methylamphetamine, methamphetamine, or amphetamine significantly increased during the pandemic, whereas MDMA and ecstasy-related presentations significantly decreased. The reason for the decrease in MDMA and ecstasy-related presentations may be because the majority of these drugs are imported into Australia, and the pandemic affected supply chains. Additionally, MDMA and ecstasy are recognized as 'party drugs' [11-12], and there were significantly fewer parties and open nightclubs in 2020 in Victoria due to strict restrictions. Conversely, methamphetamine or amphetamine is manufactured in Australia, providing easier access, and may have been substituted for other drugs with reduced accessibility. Speed is another form of methamphetamine; however, ED presentation cases of this stimulant were too small for both years; hence it was excluded in this study, as seen in Table 2.

Other Australian studies have reported a decrease in the use of methamphetamines throughout the COVID-19 pandemic. The Burnet Institute conducted a survey among 1,300 people in Melbourne, Victoria, who use and inject drugs, and reported a slight decline in the use of crystal methamphetamine during the COVID-19 pandemic, with less than five participants claiming that they wanted to use crystal methamphetamine but were unable to procure the drug [13]. Moreover, the report stated that the cost of methamphetamine had increased from $320 per gram after 29 March 2020 compared to the pre-COVID-19 price of $285 per gram [13]. In terms of drug purity, however, there was a difference in perception, as 47% of the participants reported a reduction in drug purity, but 35% claimed the drug purity to be greater than normal after March 2020 [13], which may contribute to the greater number of ED presentations reported in the current study. An Australian study that explored the short and long-term impact of the COVID-19 pandemic on Australians who regularly (at least once a month) use illicit drugs found stimulants were the most commonly used drugs during the COVID-19 pandemic, following alcohol, cannabis, and tobacco, with 41% reporting use of MDMA and 30% cocaine [14].

COVID-19 restrictions are expected to affect the health of people who use drugs as the restrictions require the practice of social isolation, which can lead to an increased risk of drug-related harms, and consequently require presentations to EDs. For example, among people who inject drugs, many needle and syringe programs may have closed or been unable to provide services and programs in response to the pandemic, putting people who inject drugs at greater risk of needle-transmitted infections [15]. Moreover, services for people who use drugs, e.g., harm reduction, drug treatment, and other services, have faced challenges, including limits to face-to-face contact and increased redeployment of resources and manpower to support COVID-19 public health efforts [3]. These are important to note as stimulant-related ED presentations are known to be complex and resource-intensive [16]. The ADAPT study found that over half (57%) of participants who regularly use drugs reported their mental health was worse than pre-COVID restrictions [14]. Over one-third (37%) of participants had accessed help from mental health services in the past month, 8% tried to access support but could not, and 3% were unable to access drug treatment over the past month [14]. A concern then is that the pandemic can exacerbate harms related to stimulant use, and ED presentation may be the only option for some people to access mental health services and drug treatment. The results of this study and others indicate that a review may be needed of how access to mental health and drug services could be improved during a public health emergency. Further research is required to identify the impact of the COVID-19 restrictions to better manage and care for ED presentations among people who use stimulants during times of crisis like the pandemic.

Limitations

The study only includes ED presentations of people who use stimulants from March 2019 to September 2019 compared to ED presentations during the 2020 lockdown periods in Victoria, from March 2020 to September 2020. The VISU bases its data on the VEMD, which holds de-identified clinical information from 39 Victorian public hospitals with a 24-hour ED [8]. The data from this study relied on ED staff identifying the stimulant used by the people presenting to the EDs of Victorian public hospitals. Therefore, it is possible that stimulant-related events may be inaccurately reported due to ED staff incorrectly identifying the stimulant used. Also, data is only from one state in Australia; therefore, the findings may not be generalizable to all other states in Australia or nationally.

## Conclusions

The findings of this study identified that during the COVID-19 pandemic in 2020, the number of methylamphetamine or methamphetamine or amphetamine-related ED presentations significantly increased while MDMA and ecstasy presentations declined. The COVID-19-related restrictions and relevant relief efforts can exacerbate harms related to stimulant uses due to the stretching of already restricted resources, particularly in areas like the EDs. Therefore, there is a need to ensure that the resources will be available if it becomes necessary to attend to the emergency needs of patients presenting to ED due to stimulant use.
